# VerdaML: an explainable ensemble machine learning framework with AI-driven interactive decision support system for biomass yield optimisation and Nitrogen-Stress Risk Classification in verrucodesmus verrucosus photobioreactors

**DOI:** 10.3389/fbioe.2026.1906898

**Published:** 2026-09-23

**Authors:** Ritu Chauhan, Zainab Sarfi, Ekampreet Soni, Vivek Kumar Gaur, Megat Zuhairi

**Affiliations:** 1 Amity Institute of Biotechnology, Amity University, Noida, India; 2 Universiti Kuala Lumpur, Kuala Lumpur, Malaysia

**Keywords:** biomass prediction, ensemble machine learning, explainable AI, lipid accumulation, microalgae, photobioreactor, sustainable bioproduction, verrucodesmus verrucosus

## Abstract

Optimisation of microalgal photobioreactor cultivation is essential for the sustainable production of bioproducts, and is challenged by the complex interaction of parameters, low experimental signals and the simultaneous maximisation of biomass productivity and lipid accumulation for biofuel production. This paper introduces VerdaML, an explainable, dual-objective machine learning framework to predict biomass yield of Verrucodesmus verrucosus and model nitrogen-stress induction risk as a proxy for lipid-inductive cultivation conditions as well as provide interaction-based decision support. Domain-informed feature engineering was used to recognise process optimality, stress, and interaction effects using an open-access dataset consisting of 1,080 photobioreactor observations. Biomass yield was predicted using robust regression and ensemble learning, while imbalance-aware classification with SMOTE was used to predict the lipid accumulation potential. The Gradient Boosting regression model yielded a near-ideal yield prediction (*R* ^2^ = 0.9867, RMSE = 0.1018 g/L, MAE = 0.0763 g/L) and a soft voting ensemble classifier achieved an accuracy of 92.1% (AUC-ROC = 0.9903) for nitrogen-stress risk classification. Five-fold cross-validation confirmed model generalisability, with Gradient Boosting achieving a mean cross-validated *R* ^2^ of 0.9842 (SD = 0.0024), consistent with the held-out test performance. Joint analysis revealed some trade-offs between fast biomass growth and lipid biosynthesis, and emphasized the drawbacks of single-objective optimization approaches. As a data-driven interactive AI framework, VerdaML makes it possible to optimise photobioreactor processes risk-consciously in adherence to the sustainable bioproduction principles recommended by the United Nations Sustainable Development Goals 7 and 9.

## 1 Introduction

The rising demand for green products as alternatives to petroleum-derived products has led to a greater interest in photosynthetic microbial cell factories for biofuels, specialty chemicals and high-value bioproducts (Ramchuran et al., 2023). Microalgae are highly productive photosynthetic microorganisms that can rapidly accumulate biomass and store lipids within their cell walls under nutrient stress conditions, making them suitable feedstock organisms for biodiesel production (Chisti, 2007; Mata et al., 2010). Thus, the optimization of the efficiency, robustness, and reliability of microalgal cultivation in photobioreactors is of great significance for the development of sustainable industrial biotechnology (Posten, 2009).

Despite the development of cultivation technology and metabolic engineering, performance of microalgal photobioreactors is highly sensitive to the operating conditions, such as irradiance, temperature, pH, dissolved oxygen, CO2 supply, and nutrient concentration (Abiusi et al., 2014; Juneja et al., 2013). These parameters are coupled in an intricate and nonlinear fashion, often yielding weak and noisy experimental signals. In such cases, traditional optimisation methods like response surface methodology and mechanistic kinetic modelling are not scalable and generalizable enough (Quinn and Davis, 2015). Machine learning (ML) has thus emerged as a potential data-driven alternative to modelling and predicting the results of microalgal cultivation (Wang et al., 2022).

Recent research on ML-based models has demonstrated improved biomass prediction and parameter optimisation in microalgal bioprocess systems, but most approaches address only the single objective of maximising biomass productivity without simultaneously modelling lipid accumulation potential, which is the primary performance indicator for biofuel applications (Syed et al., 2024). Moreover, most ML models are still restricted to offline analysis, which limits their application to real-world decision-making and industrial deployment (del Rio-Chanona et al., 2019). AI-driven interactive decision support systems and simulation frameworks offer a potentially effective avenue to counter these challenges by incorporating predictive models, data analytics, and interactive scenario analysis in a single decision-support platform (Park et al., 2021; Ras et al., 2022). Nevertheless, the development of AI-based frameworks for microalgal cultivation systems that simultaneously address biomass productivity and lipid accumulation potential has not been previously reported.

This study has three principal contributions. To begin with, we show that ensemble machine learning models combined with domain-informed feature engineering can predict microalgal biomass yield with near-ideal accuracy from limited photobioreactor sensor data. Second, we demonstrate that explicit lipid accumulation potential modelling with class imbalance correction substantially improves detection of high-yield lipid-favourable conditions. Third, we introduce VerdaML, a deployable AI-based interactive framework that allows scenario-based photobioreactor optimisation aligned with the objectives of industrial sustainability.

The suggested framework supports sustainable biofuel production from microalgal cell factories, reduces experimental burden through virtual scenario simulation, and facilitates operational choices based on data-driven decision support to contribute to clean energy pathways and resilient biomanufacturing infrastructure in accordance with the United Nations Sustainable Development Goals 7 and 9. On the whole, this article demonstrates that machine learning and AI-driven interactive decision console may be successfully combined to support sustainable production of bioproducts from photosynthetic microorganisms.

Machine learning and artificial intelligence have been progressively applied to optimise microalgal cultivation, particularly in the areas of biomass yield prediction and growth condition modelling (Imamoglu, 2024). The use of data-driven models has shown high efficacy in modelling nonlinear relationships among cultivation parameters and outcomes, providing an alternative to traditional mechanistic and statistical methods (Khaleghi et al., 2021). Studies have used Artificial Neural Networks (ANN) and Support Vector Machines (SVM) to model biomass productivity in microalgal systems using operational variables including temperature, light intensity, pH, CO2 concentration, and nutrient availability, achieving good predictive performance (De Farias Silva et al., 2022). Other AI-based optimisation models have been applied to predict biomass and lipid content in various microalgal species and found high agreement between predicted and experimental outcomes (Wu et al., 2025). Process optimisation of a photobioreactor using hybrid techniques of neural networks and evolutionary algorithms has also been investigated (Aziz et al., 2020). Later research has evolved static prediction to dynamic monitoring and control, and deep learning models are created for the non-stationary cultivation problem, such as feeding control and real-time monitoring (Igou et al., 2023). As per the reviews of AI in microalgal bioprocesses, the most commonly used input variables in the monitoring, optimisation and control applications were irradiance, pH and temperature, and machine learning, deep learning, and ensemble methods are gaining more and more importance (Syed et al., 2024).

Nevertheless, the majority of AI-based microalgal cultivation research has been framed as a single-objective problem focused on biomass maximisation, with scarce attention drawn to simultaneous modelling of lipid accumulation potential or dual-objective optimisation frameworks. Other research has combined machine learning and interactive decision support system to improve accessibility of smart bioprocess systems (Yee et al., 2025). Shortcomings of single-objective optimisation strategies including trade-offs between growth rate and lipid biosynthesis, nutrient limitation effects, and cultivation phase transitions have been noted across several studies (Hu et al., 2008; Converti et al., 2009). Ensemble learning methods have been widely used to enhance predictive robustness in bioprocess modelling, consistently outperforming single models in noisy and small-sample environments (Wang et al., 2023; Mukasine et al., 2024).

While the effectiveness of AI and ensemble learning has been demonstrated across the large body of literature on bioprocesses, there is a lack of integrated frameworks that can be used to optimise productivity while controlling the potential for lipid accumulation in an interactive AI-based platform for photosynthetic microalgal cell factories. The present study aims to fill this gap by introducing VerdaML, a dual-objective and explainable AI approach to optimize the cultivation of Verrucodesmus verrucosus in a photobioreactor.

A comparative summary of these prior single- and dual-objective microalgal ML frameworks against VerdaML is provided in Table 1.

**TABLE 1:** summarises existing machine learning frameworks for microalgal cultivation optimisation and highlights the unique contributions of VerdaML relative to prior work.

References	Objective	ML method	Best performance	Dashboard	Dual objective	SMOTE	Feature engineering
(del Rio-Chanona et al., 2019)	Biomass yield (single)	Deep learning surrogate	High (task-specific)	✗	✗	✗	✗
(Wu et al., 2025)	Biomass and lipid (separate models)	ANN, RF, SVM	Varies by study	✗	✗	✗	Partial
(Syed et al., 2024)	Biomass prediction (single)	CNN, RF, GBDT, LSTM	R^2^ > 0.95	✗	✗	✗	Partial
(Igou et al., 2023)	Real-time biomass (single)	Deep learning (LSTM)	R^2^ = 0.91	✗	✗	✗	✗
(Imamoglu, 2024)	Growth prediction (review)	ANN, SVM, RF	Review only	✗	✗	✗	✗
(Mondal et al., 2023)	Bioprocess optimisation (review)	Ensemble ML (review)	Review only	✗	✗	✗	✗
VerdaML (This study)	Biomass + N-stress risk (dual)	GB + Soft Voting Ensemble	R^2^ = 0.9867; AUC = 0.9903	✓ 5-tab	✓	✓	✓ 17 features

VerdaML, is the only reported framework combining dual-objective modelling, SMOTE-based imbalance correction, domain-informed feature engineering, and an interactive five-tab decision support system within a single deployable platform.

## 2 Methodology

### 2.1 Principle of the proposed framework: VerdaML

The cultivation of microalgae in photobioreactors is a nonlinear interaction of variables with limited experimental data availability, and requiring calibration of both biomass productivity and lipid accumulation for biofuel production. The properties make the use of traditional statistical modelling and heuristic optimisation techniques too uncertain, particularly in the context of maximizing the productivity and lipid yield. To address these issues, the proposed study presents VerdaML, an explainable, dual-objective, machine learning based AI framework for optimising Verrucodesmus verrucosus photobioreactor cultivation. VerdaML is a data-driven surrogate model that simulates photobioreactor behaviour using historical sensor data. Rather than approximating the underlying photosynthetic biochemical kinetics through mechanistic equations, the proposed framework learns statistically robust correlations between cultivation conditions and process outcomes using advanced ensemble machine learning methods. The main design concept of VerdaML is that biomass yield prediction and lipid accumulation potential classification are integrated in a single analytical framework, which allows to make informed decisions in the light of operational constraints.

VerdaML is specifically designed to address two essential challenges of microalgal bioproduction. First, it forecasts maximum biomass yield in terms of controllable process parameters, facilitating data-driven yield optimisation. Second, it predicts the probability of high lipid accumulation, which is a condition characterized by high intracellular triacylglyceride content favourable for biodiesel feedstock production, with the same operational inputs. This dual-objective strategy is similar to the industrial demand for fast biomass growth and metabolic shift to lipid biosynthesis under nitrogen limitation (Hu et al., 2008; Converti et al., 2009).

The overall architecture of the VerdaML system is shown in Figure 1. The framework starts by ingesting data from the sensors of the photobioreactor, pre-processing it and applying domain knowledge-driven feature engineering to boost the quality of the predictive signal. The models of ensemble regression and classification are then trained to predict biomass yield and detect lipid potential, respectively. Model outputs are assessed using task-specific performance metrics, and trained models are made available to users via an interactive five-tab AI-driven interactive decision support console that allows users to make predictions and assess cultivation trade-offs based on scenarios. In this interpretable and modular design, VerdaML offers a feasible decision support system for scalable and sustainable microalgal bioproduction.

**FIGURE 1:**
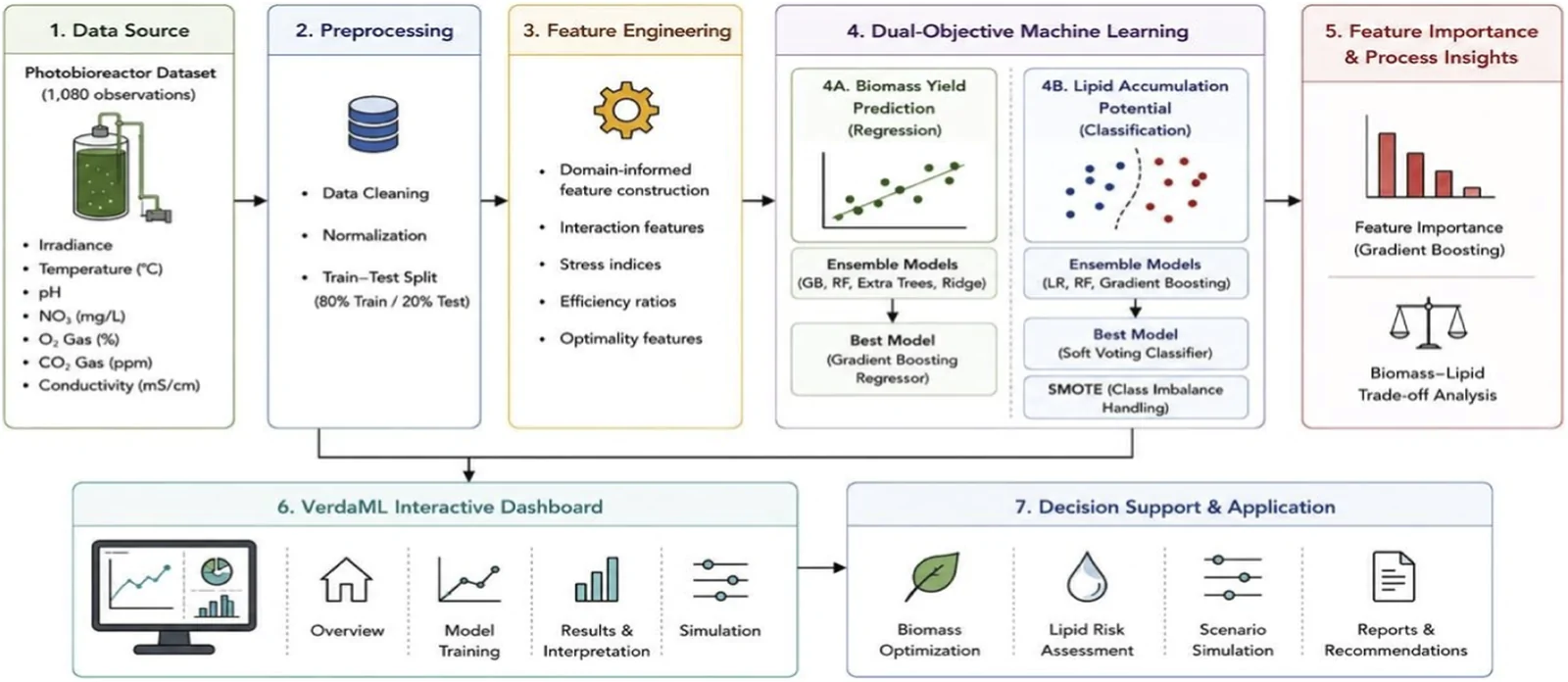
Proposed architecture of the VerdaML framework showing data processing of photobioreactor sensor data, ensemble-based biomass yield and lipid accumulation modelling, and risk-sensitive decision support using an interactive AI-driven decision support platform.

### 2.2 Description of dataset and problem formulation

The data used in this research was obtained from a publicly available open-access repository (Mendeley Data, DOI: 10.17632/c7f34t83pd.3) comprising physicochemical sensor measurements from Verrucodesmus verrucosus cultures grown in bubble column photobioreactors under controlled conditions (Hernandez-de Leon et al., 2025). This dataset contains 1,080 observations from cultivation runs in a photobioreactor, the main performance metric being biomass dry weight (g/L). The data set contains measurements of seven physicochemical variables: irradiance (Lux), nitrate concentration (NO3, mg/L), temperature (°C), pH, oxygen off-gas fraction (O2 Gas, %), carbon dioxide off-gas fraction (CO2 Gas, ppm), and electrical conductivity (μS/cm). Optical density (OD600) was also included in the raw data but was not included in any of the predictive modelling as it was considered as a direct optical measurement of biomass concentration and would cause data leakage when training the models. There were no missing values in the data set. Table 2 provides a tabular description of the features of the dataset, including variable names, descriptions, units, and bivariate correlations with the biomass target variable.

**TABLE 2:** Description of photobioreactor dataset features including variable names, definitions, units, and Pearson correlations with biomass yield, with identification of primary response variables.

Feature name	Description	Unit	Corr. W/Biomass
Irradiance	Light intensity supplied to photobioreactor culture	Lux	+0.192
NO3	Nitrate concentration in culture medium	mg/L	−0.966
Temperature	Culture temperature	°C	+0.313
pH	pH of culture broth	—	+0.776
O2 Gas	Oxygen off-gas fraction	%	−0.979
CO2 Gas	Carbon dioxide off-gas fraction	ppm	−0.951
Conductivity	Electrical conductivity of culture medium	μS/cm	+0.809
OD600 (excluded)	Optical density — excluded to prevent data leakage	—	−0.211
Biomass (g/L) (regression target only, not used as an input feature)	Dry weight — primary continuous response variable	g/L	1.000

According to this data, a modelling problem is formulated as a dual-objective supervised learning task. The first objective aims at predicting biomass yield by determining the maximum biomass dry weight (g/L) as a result of photobioreactor operating conditions, which is a regression problem. The second objective is to predict whether a given set of operating conditions is associated with high lipid accumulation potential, formulated as a binary classification task. The continuous variable for biomass was converted to a binary lipid accumulation potential target by assigning observations that were equal or greater to the 65th percentile (2.29 g/L) to a high lipid-inductive potential (nitrogen-stress risk index) class, using the known biology of lipid induction in oleaginous microalgae under nitrogen stress (Hu et al., 2008; Converti et al., 2009; Breuer et al., 2012; Klok et al., 2013). This classification is not a direct measurement of lipid content, but rather a nitrogen-stress risk index. Biological rationale is clear: in oleaginous microalgae, the depletion of nitrogen leads to a metabolic switch from protein synthesis to carbon storage as triacylglycerides, which is likely to happen at high cell densities where nitrate has been extensively used (Hu et al., 2008; Converti et al., 2009). The 65th percentile threshold (2.29 g/L) was chosen to represent those cultivation runs where low NO3 and high conductivity, the two best predictors of biomass in this data set are aligned with conditions that are likely to induce lipids. Future validation against direct GC-MS fatty acid methyl ester quantification is recommended to confirm the biological validity of this classification. Both objectives are optimised concurrently, enabling simultaneous assessment of biomass productivity and nitrogen-stress induction risk. The methodology enables scenario analysis, where the conditions within the photobioreactor could be manipulated to determine the impact on productivity and lipid potential, which enables flexibility to identify the operating range suitable to scalable and sustainable production of biodiesel feedstocks.

### 2.3 Data pre-processing and feature engineering

Raw photobioreactor sensor data frequently contain weak individual predictive signals due to biological variations and the characteristically limited signal-to-noise ratio of individual sensor measurements. In order to make the proposed framework robust and reproducible, a systematic data pre-processing and feature engineering pipeline was applied before model training.

The dataset was complete, with no missing entries requiring imputation. Prior to training, z-score standardisation (StandardScaler) was applied to all continuous input variables, producing a zero-mean, unit-variance distribution for each feature. This step prevents larger-valued variables from dominating distance- or gradient-based optimisation. Standardisation parameters were fitted on the training subset alone and then applied to the test subset to prevent data leakage.

Apart from the classic pre-processing, domain-informed feature engineering was performed on a large scale to boost the weak predictive signals typical of the photobioreactor sensor data. Synergistic interactions between selected operational variables were captured by creating interaction features: Irradiance × pH, NO3 × O2 Gas, Temperature × pH, CO2 × Irradiance, and Conductivity × pH, as these interactions are known in the physiology of microalgae and their roles in photosynthetic activity and metabolic performance (Abiusi et al., 2014; Juneja et al., 2013). Stress indices were built to capture the deviations from ideal operating conditions: NO3 stress (normalised deviation from mean nitrate), pH stress (absolute deviation from pH 7.5) and temperature stress (absolute deviation from 22 °C). Efficiency ratio features were derived to represent normalised process behaviour: irradiance-to-nitrate ratio and O2-to-CO2 ratio, the latter being a direct indicator of net photosynthetic gas exchange.

The rationale behind this domain-informed feature engineering approach is that photobioreactor sensor datasets are inherently weak-signal in nature, with individual variables having only limited predictive potential in isolation (Syed et al., 2024). Derived features are an effective way of introducing domain knowledge to learning algorithms, improving the generalisation capability and decreasing the sensitivity to experimental noise. Table 3 summarises the engineered feature categories and their roles in the modelling pipeline, and their relative significance in the prediction of biomass yield is discussed in the Results section.

**TABLE 3:** Engineered feature sets and their functional contributions to signal improvement, biological process representation, and model performance.

Feature category	Description
Optimality features	Quantify proximity to empirically favourable operating ranges
Stress indices	Capture cumulative deviation from stable process conditions
Interaction features	Encode coupled effects between key process parameters
Efficiency ratio features	Represent normalised resource utilisation and metabolic balance

### 2.4 Machine learning models and training strategy

The biomass productivity and lipid accumulation potential of the photobioreactors were modelled using a variety of machine learning algorithms such as regularised linear models, robust regression models, tree-based ensembles, and ensemble meta-modelling approaches. This diversity was chosen to assess complementary learning behaviours, improve generalisation in sparse data scenarios and minimise overfitting in weak-signal photobioreactor data.

#### 2.4.1 Biomass yield prediction models

- Ridge Regression served as the regularised linear reference model for biomass yield prediction. By applying an L2 penalty to the regression coefficients, Ridge regression limits the influence of correlated inputs, producing reliable estimates across datasets where sensor variables are correlated, as is typical in photobioreactor cultivation.
- Bayesian Ridge Regression reframes ridge regression in probabilistic terms by assigning prior distributions to the model coefficients rather than treating them as point estimates. The regularisation strength is learned from the data, making the model adaptive to sample size and improving robustness when working with limited photobioreactor observations.
- Huber Regression was selected to handle the extreme observations that commonly arise in experimental cultivation data. The model applies a squared penalty to small residuals and switches to a linear penalty above a threshold, limiting the influence that unusual biomass measurements have on the fitted regression surface.
- Gradient Boosting Regression trains a series of decision trees sequentially, with each successive model correcting the residuals of its predecessor. The iterative learning method allows for the capture of nonlinear relationships between operating parameters of the photobioreactor and the biomass yield at a fine level of detail (Mondal et al., 2023).
- Extra Trees Regression extends the randomisation of standard tree ensembles by sampling split thresholds uniformly at random rather than searching for the locally best split. The added stochasticity further lowers individual tree variance, which is beneficial when the feature space is high-dimensional after engineering.
- Random Forest Regression combines predictions from many decision trees, each fitted to a bootstrapped sample of the training data using a randomly selected feature subset at each node. Averaging across trees reduces prediction variance while retaining the capacity to capture nonlinear interactions between photobioreactor variables (Wang et al., 2023).
- Voting Ensemble aggregates the numerical outputs of the Random Forest, Gradient Boosting, and Extra Trees regressors with equal weights. Models with different inductive biases tend to produce complementary errors, so their average typically achieves better generalisation than any constituent model alone (Wang et al., 2023; Mukasine et al., 2024).

#### 2.4.2 Nitrogen-stress risk classification models

A set of supervised classification algorithms was used to detect and predict conditions associated with high nitrogen-stress induction risk, used as a proxy for lipid-inductive cultivation states, during photobioreactor cultivation. Models were chosen to ensure they were interpretable and had the ability to learn nonlinearly, which is necessary to capture rare, but commercially important, lipid-inductive cultivation states (Hu et al., 2008; Converti et al., 2009).

- Logistic Regression provided the linear reference point for lipid potential detection. Its sigmoid output produces calibrated class probabilities that allow direct comparison with the more flexible ensemble classifiers, making it straightforward to quantify the performance gain from introducing nonlinearity.
- Random Forest Classification resolves nonlinear class boundaries by aggregating class votes from many bootstrapped decision trees, each trained on a different random feature subset. This architecture handles the heterogeneous sensor measurements in photobioreactor data well and is relatively resistant to noise, making it suitable for detecting the rare high lipid-potential conditions.
- Gradient Boosting Classification was applied for its sensitivity to subtle patterns in the data. Trees are grown one at a time, with each fitted to the residual errors of the current ensemble, directing model capacity toward the observations that are most difficult to classify correctly. This makes the approach effective when lipid induction depends on the interaction of multiple operating parameters simultaneously.
- Soft Voting Ensemble averaged the posterior class probabilities from Logistic Regression, Random Forest, and Gradient Boosting rather than combining hard label votes. Probability averaging exploits the complementary inductive biases of the three models, and has been shown to be particularly beneficial for minority-class detection—here the high lipid-potential observations that represent the target operating conditions for biodiesel feedstock harvest (Chawla et al., 2002).

The combination of these classification models provides complementary views of lipid accumulation potential that can be used to reliably identify high lipid-favourable conditions and support risk-conscious microalgal cultivation management (Yee et al., 2025).

#### 2.4.3 Training strategy and evaluation

Data were partitioned into training (864 observations, 80%) and test (216 observations, 20%) subsets using stratified sampling to preserve the class distribution of the lipid accumulation target in both splits. Regression performance was measured using *R* ^2^, RMSE (g/L), and MAE (g/L). For classification, accuracy, precision, recall, F1-score, and AUC-ROC were computed on the held-out test set; recall received particular emphasis because false negatives represent missed opportunities to detect commercially valuable high lipid-potential conditions.

### 2.5 Class imbalance handling and dual-objective modelling

The photobioreactor dataset is inherently imbalanced—observations classified as low nitrogen-stress (low lipid-inductive) substantially outnumber those classified as high nitrogen-stress (high lipid-inductive) (65% vs. 35%). This skewness represents a major hurdle for supervised learning, because classifiers trained on skewed data are prone to overfitting the majority class and perform poorly on the minority class, which is operationally important but rare (Chawla et al., 2002).

Class imbalance was addressed through the Synthetic Minority Over-sampling Technique (SMOTE) (Chawla et al., 2002). Rather than duplicating existing minority-class records, SMOTE generates new synthetic observations by linearly interpolating between a minority-class sample and one of its nearest neighbours in feature space. This produces a richer and more varied representation of high lipid-potential conditions, which sharpens the classifier’s decision boundary for this class. SMOTE was applied exclusively within the training fold; the test set retained its original class distribution so that evaluation metrics reflect real-world operating conditions. After resampling, the classification training set comprised 1,126 observations equally split between the two classes.

The application of SMOTE in the proposed framework was selective and was used only in the lipid accumulation classification task. The original non-resampled data was used for training biomass yield prediction models to avoid artificial distortion of the continuous target variable because of the natural variance of the continuous productivity measurements. This selective use of imbalance correction reflects the dual-objective design philosophy of VerdaML. Optimisation of the photobioreactor is not a single objective problem, but rather the explicit separation of productivity optimisation and the modelling of the lipid risk allows for the use of methods that are suitable for the learning task. To validate this design choice, the Gradient Boosting regression model was trained on both the original unbalanced data and a SMOTE-resampled version; results were near-identical (original: *R* ^2^ = 0.9867, RMSE = 0.1018; SMOTE: *R* ^2^ = 0.9861, RMSE = 0.1026), confirming that SMOTE provides no benefit for continuous regression targets.

### 2.6 Model evaluation and performance assessment

Performance metrics used in model evaluation of biomass yield prediction and lipid accumulation potential detection were computed independently using task-appropriate metrics to assess both predictive accuracy and operational utility.

For the regression task with the objective of maximum biomass dry weight (g/L), three complementary metrics were calculated. The coefficient of determination (*R* ^2^) is a measure of the overall predictive power of the model, representing the percentage of variance in biomass yield accounted for by the model. Root Mean Squared Error (RMSE, g/L) measures the mean magnitude of prediction errors with greater sensitivity to large deviations due to quadratic weighting. Mean Absolute Error (MAE, g/L) provides a scale-interpretable, outlier-robust measure of average prediction error. Together these metrics assess both predictive power and practical accuracy.

For the lipid accumulation potential classification task, five evaluation metrics were computed to account for class imbalance and the asymmetry of error costs. The overall accuracy is the measure of correctness in classification. Precision quantifies the positive predictive value for high lipid-potential predictions. Recall refers to the sensitivity to true high lipid-potential conditions and is of primary operational importance because missed detections represent lost opportunities for harvest of biodiesel feedstock. F1 score is a balanced measure of precision and recall. AUC-ROC is a threshold independent assessment of the lipid potential detection capability, as it quantifies the discriminative ability across all decision thresholds. Stratified train-test splitting was performed and all metrics were computed on the held-out test set.

### 2.7 Explainability and interpretability

The predictive accuracy alone is not enough to guide the operation of a photobioreactor, as model outputs may lead to wrong recommendations in complex cultivation conditions that may result in productivity or yield losses. To compensate for this, VerdaML has built in systematic explainability mechanisms to identify the most important operational parameters and engineered features affecting the biomass yield and lipid accumulation potential of the model.

Feature importance scores derived from the best-performing Gradient Boosting regression model quantify the average contribution of each variable to the reduction of biomass prediction error across all trees in the ensemble (Imamoglu, 2024; Mondal et al., 2023). This analysis moves beyond black-box prediction to provide insight into process parameters and engineered features that play the most critical role in cultivation performance of the photobioreactor. For yield prediction, interpretability analysis can be used to determine operational variables and engineered features that most strongly influence biomass productivity, helping to identify optimal operating regimes.

The framework separates interpretability analysis based on the two modelling objectives,productivity and lipid potential meaning that optimisation recommendations are informed both by yield enhancement and by lipid induction risk. This dual interpretability layer serves as a facilitator in bridging the predictive modelling and actionable process understanding gap, fostering trust and transparency in the optimisation of the photobioreactor through AI and Machine Learning.

### 2.8 Decision support interface implementation

To put the VerdaML framework into operation, we created an interactive decision support system, a web-based tool with five tabs: data overview, model training documentation, performance evaluation and interactive scenario simulation all in one comprehensive platform. This decision support simulation platform was created using HTML5, CSS3 and JavaScript with Chart. js for interactive data visualisation.

Each of the functional tabs of VerdaML correspond to a different phase of the modelling and the decision support workflow.

- Home tab: Entry point of the system. Displays indicators to communicate system status and for model readiness. Overview of the reference framework. Summary of correlations between photobioreactor sensor variables and biomass yield. Also communicates key dataset characteristics.
- Overview tab: Provides statistics for the Photobioreactor Dataset including visualisation of distribution of Biomass Yield, ranges of values of photobioreactor sensor variables, and a table assessing quality of features which indicates correlational strength and flags variables which need to be methodologically addressed.
- Train Models tab: The complete machine learning pipeline is documented, including regression and classification algorithms, SMOTE resampling procedure, and an informed feature engineering approach. This documentation provides transparency into the model construction methodology.
- Results tab: Provides comparative model performance tables, highlighting the top-performing models, feature importance bar charts showing the ranked contribution of all 17 engineered and raw features, and an *R* ^2^ comparison visualisation across all regression models.
- Simulation tab: Constitutes the primary interactive decision-support interface. Users have the option of adjusting all seven photobioreactor operational parameters using interactive slider controls, and they will receive real-time predictions of biomass yield (g/L), and lipid accumulation potential; displayed using different colours so that users can quickly identify whether they are operating in a high- or low-lipid-potential regimes, as well as receive recommendations for optimising their processes based on the expected results that will be produced by these scenarios. Prediction outputs are generated by the fully-trained Gradient Boosting regression model and Soft Voting Ensemble Classifier; all tree structures of these models are embedded in VerdaML to ensure that numerical results from scenario outputs are consistent with offline model evaluation results displayed in Tables 4 and 5.

**TABLE 4:** Comparative evaluation of biomass yield regression models on the held-out test set (n = 216).

Model	*R* ^2^	RMSE (g/L)	MAE (g/L)
Ridge Regression	0.9691	0.1550	0.1205
Bayesian Ridge	0.9693	0.1545	0.1200
Huber Regression	0.9677	0.1584	0.1186
Random Forest	0.9858	0.1051	0.0702
Gradient Boosting ★	0.9867	0.1018	0.0763
Extra Trees	0.9835	0.1132	0.0698
Voting Ensemble	0.9865	0.1025	0.0697

R^2^: coefficient of determination; RMSE: root mean squared error (g/L); MAE: mean absolute error (g/L). ★ indicates the best-performing model (highest R^2^, lowest RMSE and MAE)

**TABLE 5:** Classification performance metrics after SMOTE balancing, summarising accuracy, precision, recall, F1-score, and AUC-ROC for lipid accumulation potential detection models.

Model	Accuracy	Precision	Recall	F1-score	AUC-ROC
Logistic Regression	0.9398	0.8971	0.9286	0.9127	0.9843
Random Forest	0.9213	0.8827	0.9091	0.8944	0.9860
Gradient Boosting	0.9213	0.8944	0.9091	0.8944	0.9877
Voting Ensemble ★	0.9213	0.8827	0.9481	0.9141	0.9903

“Gradient Boosting Regression trains a series of decision trees sequentially, with each successive model correcting the residuals of its predecessor. ★ indicates the best-performing model (highest AUC-ROC).

The modularity of VerdaML allows engineers and researchers to use it for either evaluating models or interactive scenario-based process optimisation without having to perform the complete sequence of operations needed to physically test the (PBR) for each candidate operating condition.

## 3 Results

### 3.1 Dataset preprocessing results

Data from 1,080 observations of photobioreactors were used for this study. Within the entire dataset, 864 samples were used to train/develop the models and the remaining 216 were established as independent test samples. By analysing the data, it was noted that there was a much more uniform range of biomass productivity across the dataset with dry weight values ranging from 0.12 g/L to 3.33 g/L with an average (mean) biomass value of 1.67 g/L. The average operating conditions during photobioreactor cultivation runs comprised irradiance of 141.4 Lux, pH of 7.70, temperature of 22.4 °C, O2 Gas of 9.38%, CO2 Gas of 642.1 ppm, NO3 of 93.4 mg/L, and conductivity of 280.1 μS/cm, confirming that experiments were carried out in biologically feasible operating conditions.

The dataset contained no missing values, and imputation was therefore not performed. Prior to model training, all continuous input features were standardised using z-score normalisation to ensure equal weighting across variables with differing numeric ranges. Examination of the binary lipid accumulation potential target revealed that 378 observations (35.0%) corresponded to high lipid potential conditions and the remaining 702 (65.0%) to low lipid potential, yielding a moderate class imbalance consistent with the biological reality of oleaginous microalgal cultivation, where nitrogen-limited, lipid-inductive conditions represent a minority of the total operational parameter space (Hu et al., 2008; Converti et al., 2009).

### 3.2 Feature engineering results

The data from photobioreactor sensors are known to exhibit weak signals from individual features and no single operational parameter can explain the differences in biomass productivity observed from the combination of operational parameters on its own. Therefore, to maximise the occurrence of meaningful patterns and to embed domain knowledge into the learning process on a very large scale, domain informed feature engineering was employed.

Beginning with seven sensor variables, 10 additional engineered features were derived through interaction, stress index and efficiency ratio derivation resulting in a total of 17 features. Interaction features described the synergistic relationship between those key cultivation parameters (i.e., O2, CO2, and nutrients). Stress index features described the extent to which operational parameters have deviated from empirically favourable operating conditions. Efficiency ratio features described the normalised equilibrium between resource utilisation and gas exchange.

The value of engineered features used to model performance is illustrated through the feature importance analysis of the trained Gradient Boosting model. As seen in Figure 2, engineered composite features have greatly outperformed raw sensor variables in predictive contribution; the O2/CO2 ratio (19.5%), NO3 × O2 interaction (15.8%) and NO3 stress index (8.1%) combined to equal greater than the contribution from all raw variables except for NO3 alone (35.7%). The results highlight the importance of domain informed signal amplification when modelling complex photobiological systems using limited sensor data. Table 3 presents a unified summary of the engineered feature categories and their operational functions in the modelling pipeline.

**FIGURE 2:**
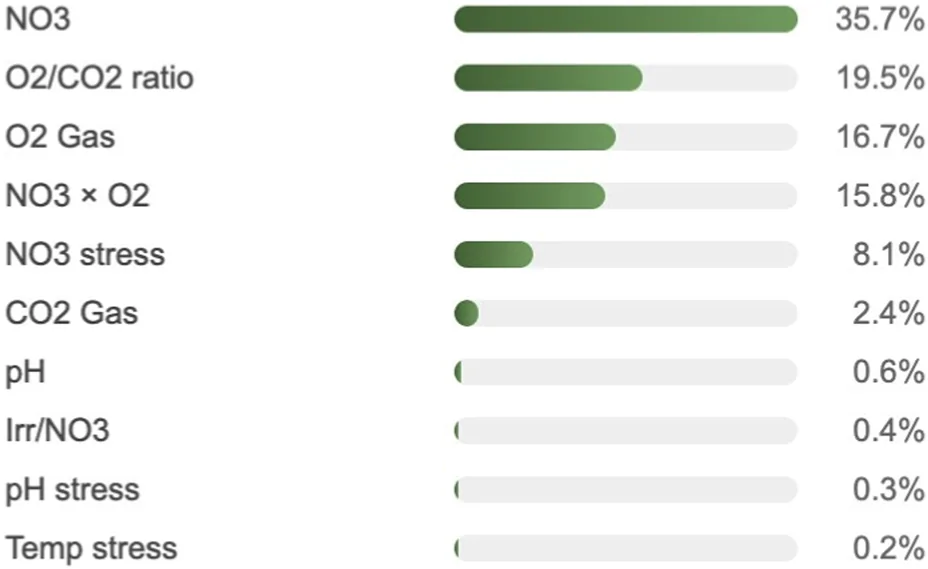
Gradient Boosting feature importance ranking showing that engineered interaction and stress features are more predictive of biomass yield compared to most individual raw sensor variables.

### 3.3 Class imbalance analysis and SMOTE results

A major obstacle to supervised classification when dealing with photobioreactor datasets is the imbalance between the two classes (i.e., low potential and high potential). In the original dataset used to classify lipid accumulation potential, 65% of observations had low lipid potential and 35% had high lipid potential. This skew in class distribution allows classifiers to learn more accurately how to classify low lipid-potential observations compared with high lipid-potential observations because they are trained on data that is biased toward one of the two classes. Models trained on skewed data tend to favour the majority class and perform poorly on the rare but commercially important high lipid potentials (Chawla et al., 2002).

To deal with this problem, SMOTE oversampling was applied only to the lipid accumulation classification training subset. As a result, the effective sample size of the classification training set was increased to 1,126 samples (563 low lipid potential and 563 high lipid potential) because the SMOTE process created new low lipid potential samples based on existing low lipid potential samples that are similar to existing high lipid potential samples. More accurate and AUC-ROC discriminative capabilities for high lipid potential detection were achieved using ensemble and tree-based classifiers following the SMOTE process (Wang et al., 2023; Mukasine et al., 2024), as shown in Figure 3, which provides evidence that the application of imbalance correction to the lipid accumulation classification training subset is necessary for meaningful lipid potential modelling.

**FIGURE 3:**
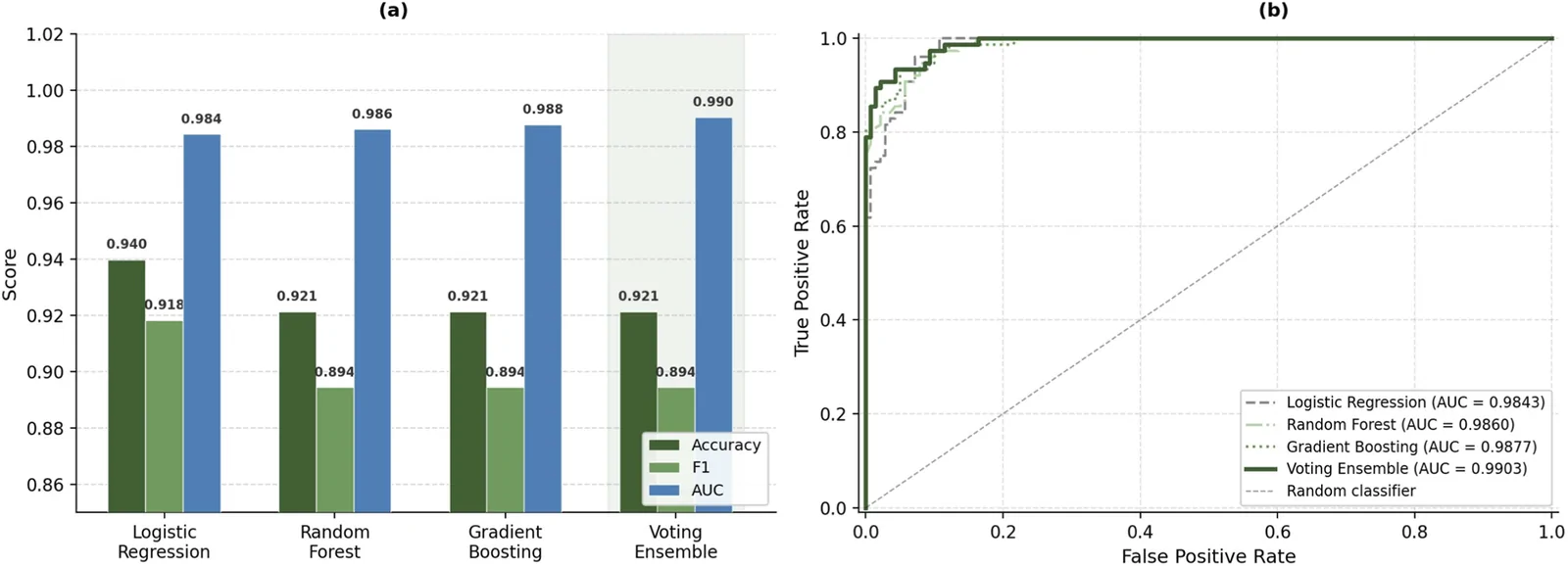
Comparison of lipid accumulation potential classification models post-SMOTE, showing higher accuracy and AUC-ROC for ensemble and tree-based classifiers than the logistic regression baseline. **(a)** Classification performance after SMOTE balancing **(b)** ROC curves - Lipid accumulation potential.

For the biomass yield regression model, the original class distribution for lipid potential was maintained. This preservation of the original distribution provided an accurate representation of the variation in continuous productivity measures, and thus prevented regression target variables from being artificially altered, making the modelling work accurately and faithfully represent the behaviour of the photobioreactor by only applying imbalance correction to the lipid accumulation classification training subset as necessary.

### 3.4 Biomass yield prediction model performance

Multiple regression models were evaluated for their predictive capacity on biomass dry weight at varied photobioreactor operating conditions. Performance was assessed using *R* ^2^, RMSE (g/L), and MAE (g/L) on the held-out test set.

Gradient Boosting regression demonstrated the best overall performance with *R* ^2^ = 0.9867, RMSE = 0.1018 g/L, and MAE = 0.0763 g/L, with actual and predicted biomass values shown in Figure 4, indicating the closeness between actual and predicted biomass values with respect to the y = x line. Other linearised models included Bayesian Ridge (*R* ^2^ = 0.9693) and Ridge Regression (*R* ^2^ = 0.9691), which also performed well, and demonstrated that domain-informed feature engineering was successful in linearising a large portion of the underlying biological non-linearity of the dataset. Huber Regression (*R* ^2^ = 0.9677) provided a good combination of linear efficiency and robust against outliers. The Voting Ensemble achieved a close performance to Gradient Boosting (*R* ^2^ = 0.9865), which supports the effectiveness of the ensemble weighting methods in predicting biomass. The comparison between all models, in terms of *R* ^2^, is presented in Figure 5. A summary analysis of all regression model performance is shown in Table 4. The models were validated against unseen observations using 5‐fold cross‐validation and z-score normalisation was applied independently within each fold. To verify that the engineered features contributed predictive value, an ablation study was conducted comparing model performance on raw sensor inputs (7 features) against the full engineered feature set (17 features). Linear models improved by 0.001–0.0012 *R* ^2^ with engineering, while tree-based models maintained equivalent performance, confirming that feature engineering adds mechanistic interpretability without sacrificing accuracy (Figure 6). The Gradient Boosting model produced the highest cross‐validated *R* ^2^ value (0.9842, SD = 0.0024) compared to the *R* ^2^ value of the test set (0.9867). The low standard deviation across folds and the negligible gap between cross-validated and test set performance confirm that the model generalises reliably to unseen observations and is not overfitting the training data. The cross-validation workflow, confirming independent standardisation within each fold, is illustrated in Figure 7.

**FIGURE 4:**
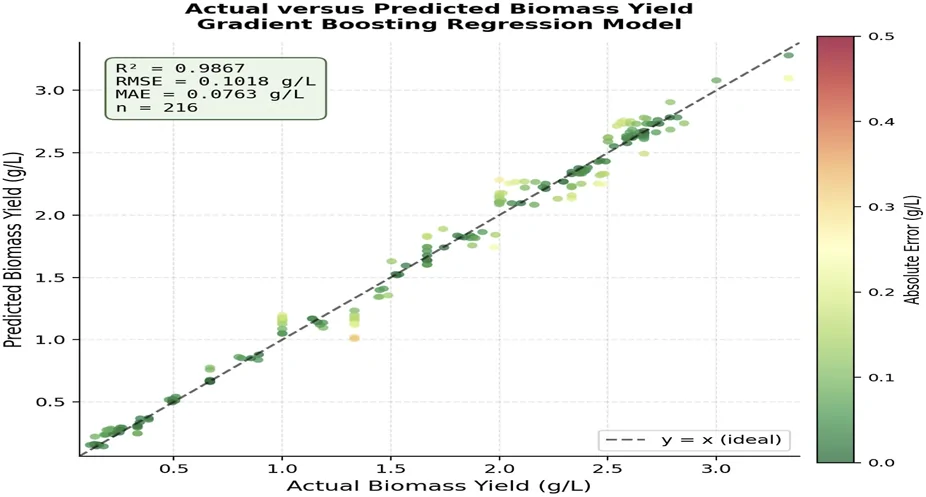
Actual versus predicted biomass yield from the Gradient Boosting model showing near-ideal correspondence to the y = x line with low prediction error.

**FIGURE 5:**
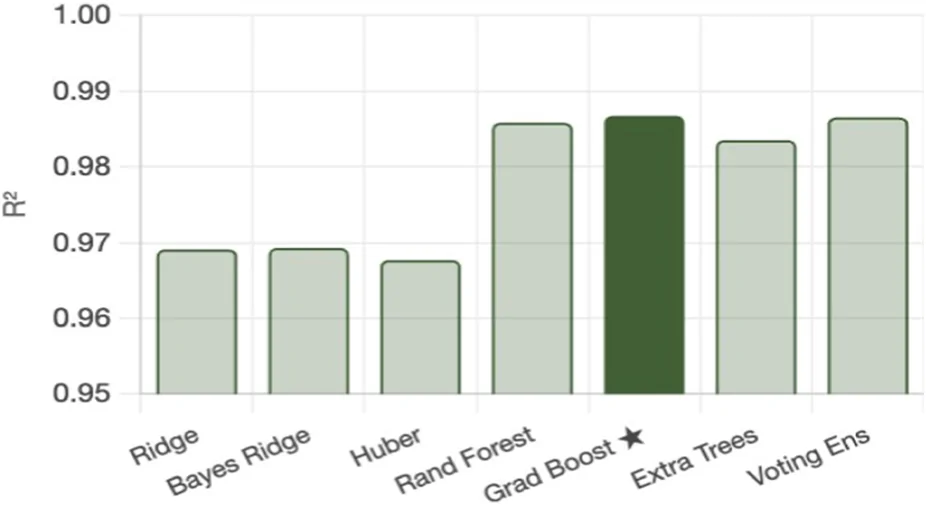
Comparative *R* ^2^ scores of regression models, with Gradient Boosting achieving the highest predictive performance.

**FIGURE 6:**
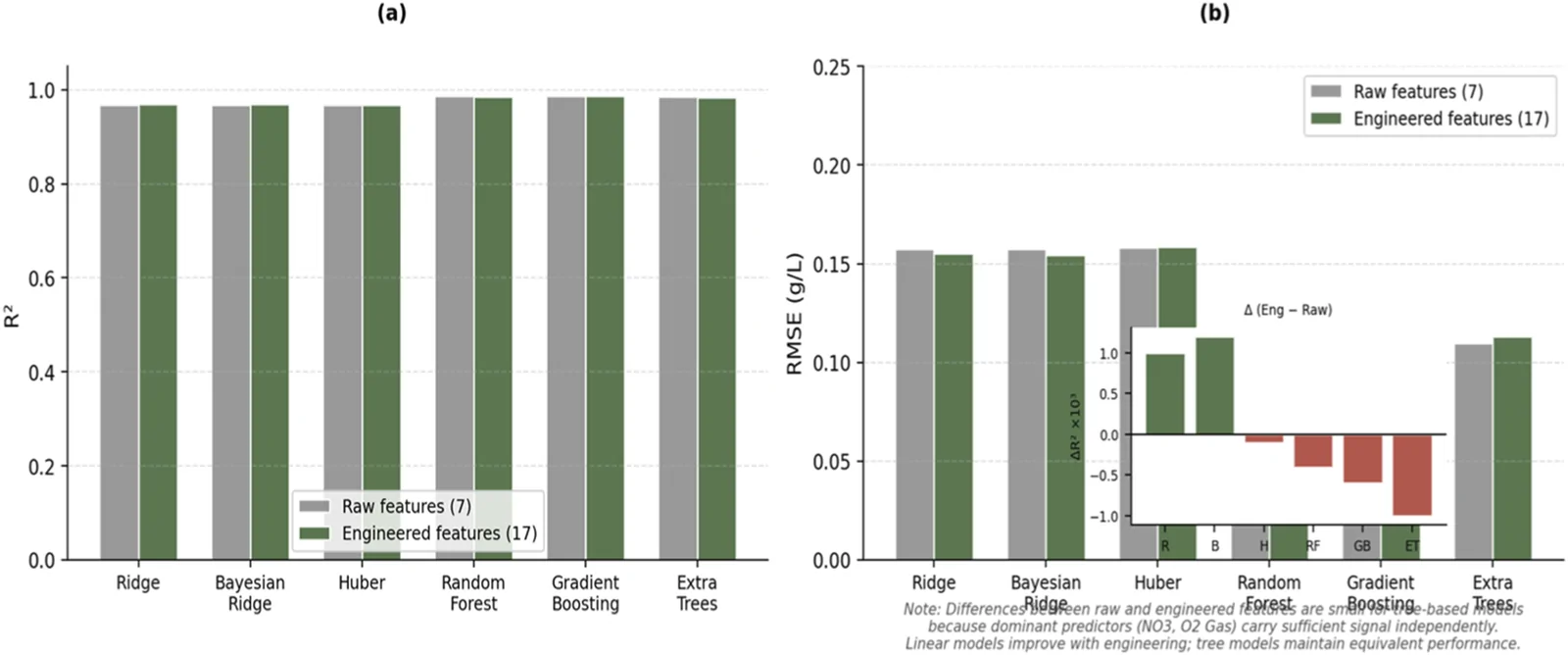
Feature engineering ablation study comparing Gradient Boosting regression performance with raw sensor variables (7 features) versus domain-engineered features (17 features). **(a)** R^2^ comparison Raw vs. engineered features **(b)** RMSE comparison Raw vs. engineered features.

**FIGURE 7:**
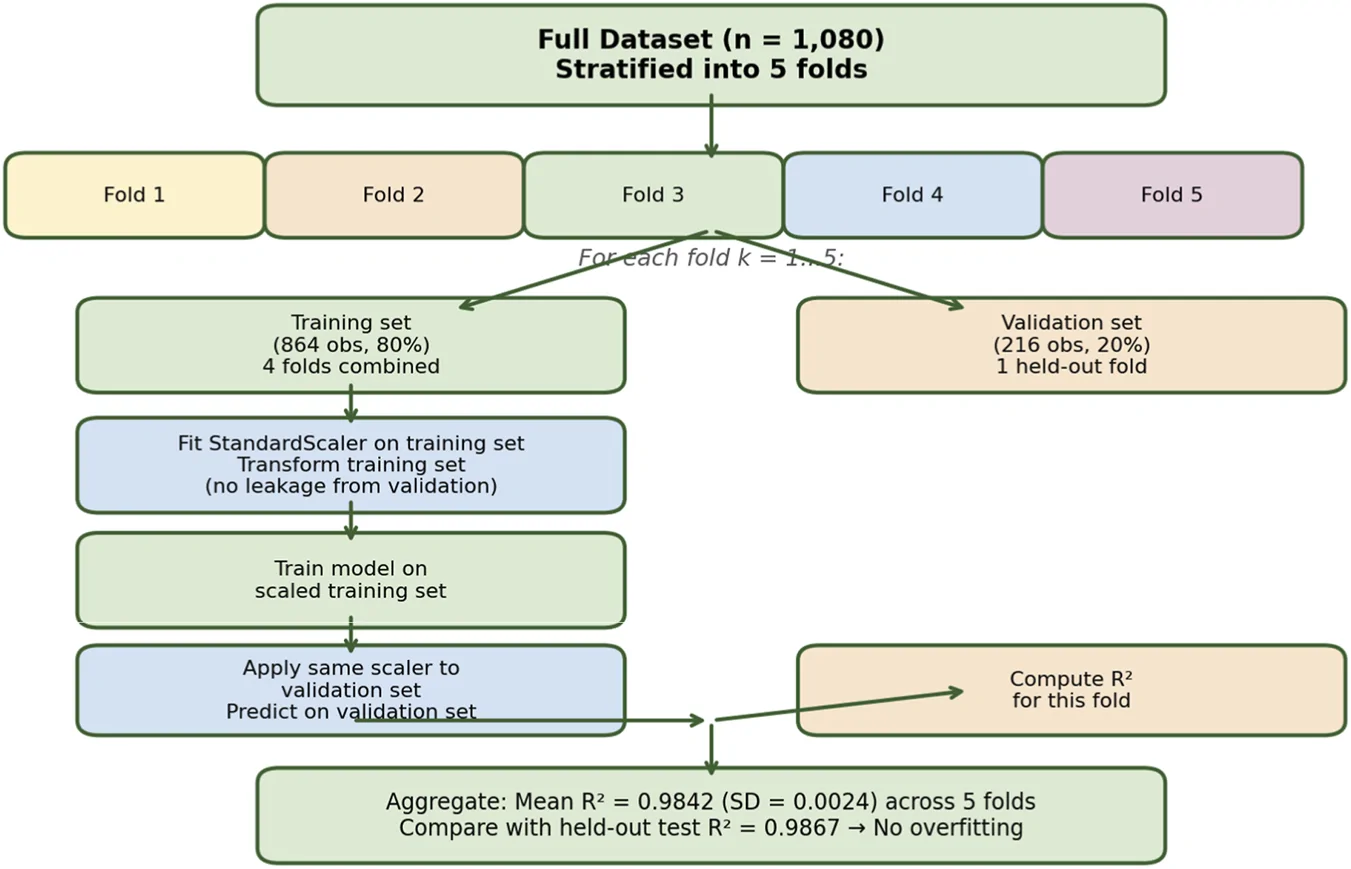
Five-fold cross-validation workflow showing independent z-score standardisation within each fold to prevent data leakage.

### 3.5 Performance of lipid accumulation potential models

To evaluate overall classification performance (accuracy, precision, recall, F1-score and AUC-ROC) and sensitivity to lipid-favourable conditions, lipid accumulation potential models were evaluated after SMOTE based training set balancing. Results revealed that ensemble classifiers consistently outperformed logistic regression baseline models.

The best overall performance was achieved with the Soft Voting Ensemble classifier (Accuracy = 92.1%, Precision = 88.3%, Recall = 94.8%, F1 = 91.4%, AUC-ROC = 0.9903). Gradient Boosting achieved the highest AUC-ROC among individual models (AUC-ROC = 0.9877), and Random Forest performed comparably (AUC-ROC = 0.9860), demonstrating the capability of both ensemble and tree-based methods to capture non-linear boundaries of the lipid potential classes. Logistic Regression achieved the highest accuracy (94.0%) due to its conservative predictions favouring the majority class, with AUC-ROC of 0.9843, indicating partial linear separability of the feature space but lower sensitivity to high lipid potential conditions when compared to the ensemble classifiers. The ensemble approach decreased variance and improved resistance to class imbalance by pooling probabilistic outputs across different classifiers, thus making the ensemble method especially useful in detecting high lipid potential minority class instances. These results support that both diversity in models and explicit correction for imbalance are critical components for successfully detecting lipid accumulation from photobioreactor datasets. Table 5 presents the complete classification performance metrics.

It is possible to evaluate trade-offs between biomass productivity and lipid accumulation potential with a dual-objective strategy that allows testing both regression and classification model performance simultaneously across conditions. In particular, several operational regimes that support high biomass production are also found to have a high potential for lipid accumulation, especially under low-nitrate, high pH, and high-conductivity conditions representing nitrogen-limited lipid synthesis in oleaginous microalgae (Hu et al., 2008; Converti et al., 2009). Conversely, when operating under elevated nitrate conditions, biomass accumulation was lower and the capacity for lipid accumulation was suppressed, demonstrating the basic antagonism between nitrogen availability and activation of the lipid biosynthesis pathway. The combined analysis confirms the limitations of single-objective optimization and shows the operational value of the dual-objective VerdaML framework (Han and Zhang, 2020; Banerjee and Mukhopadhyay, 2023).

To visualise the complete trade-off landscape between biomass productivity and nitrogen-stress induction, a Pareto front analysis was performed by generating model predictions across a grid of NO3 (23–244 mg/L) and pH (6.5–9.5) values while holding remaining parameters at their dataset means (Figure 8). The Pareto front confirms that high-biomass operating conditions (elevated NO3, low pH) consistently coincide with low nitrogen-stress induction probability, while nitrogen-depleted, high-pH conditions favour lipid induction at the cost of lower biomass yield, quantitatively validating the dual-objective design of VerdaML.

**FIGURE 8:**
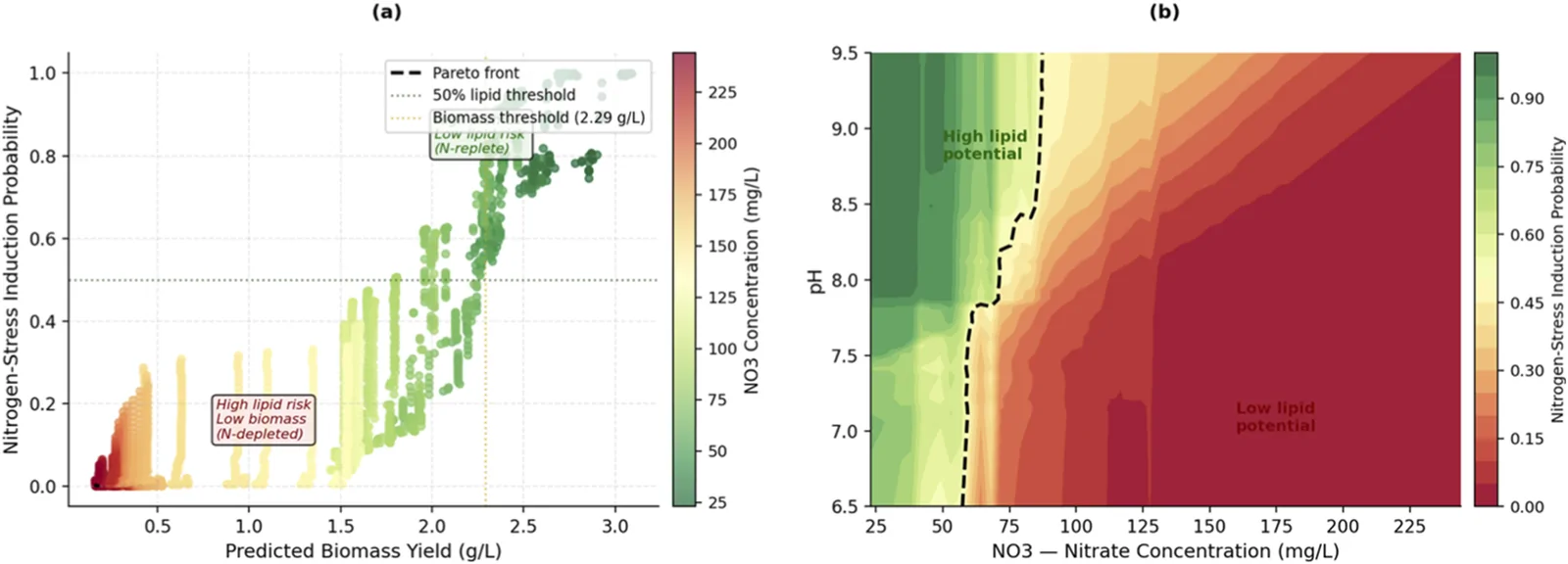
Pareto front analysis of predicted biomass yield versus nitrogen-stress induction probability across NO3 and pH operating conditions, showing the fundamental trade-off between biomass productivity and lipid-inductive cultivation states. **(a)** Biomass-lipid trade landscape with pareto front **(b)** Nitrogen-stress probability across NO3-pH operation space.

### 3.6 VerdaML and simulation results

The trained predictive workflow developed within VerdaML has been deployed as a user-friendly, interactive decision support system with a five-tabbed interface and an AI-supported predictive tool to facilitate decision-making through dual-objective optimization of photobioreactors. Within VerdaML, one can receive a complete overview of the data used in model development and performance assessment, as well as real-time scenario testing capabilities. Figure 9 illustrates the Home page of the VerdaML decision support system displaying overall system status, model readiness indicators, and key data set characteristics used in modelling. The Home page provides the first access point to the VerdaML system and provides users with a clear picture of the availability of the VerdaML system.

**FIGURE 9:**
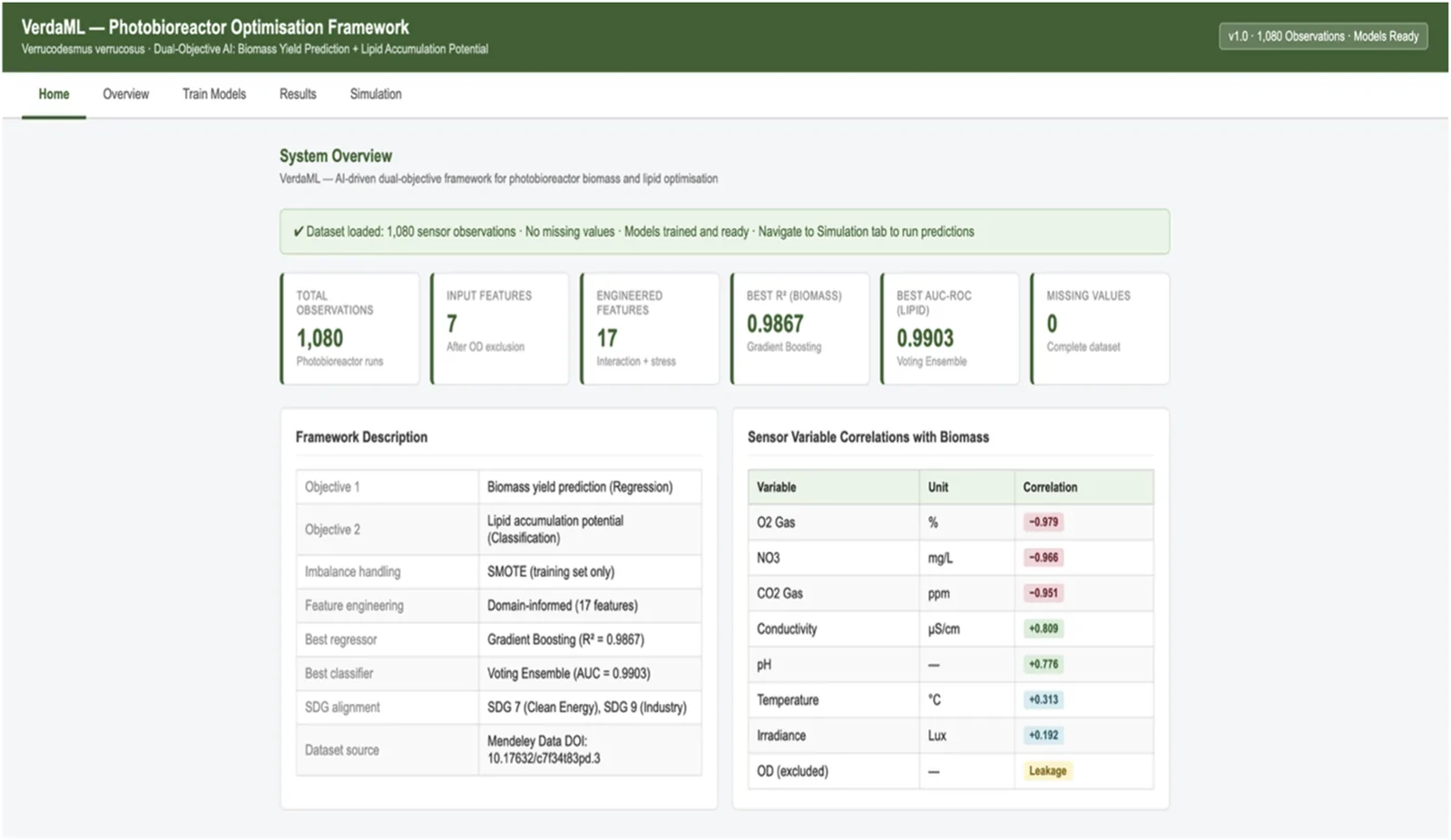
Home tab of the VerdaML decision support system showing system status, model readiness, and overview of the photobioreactor framework and dataset summary.

The Overview Tab, Figure 10, contains statistical summaries of the photobioreactor dataset. Summary statistics include the distribution of biomass yield, the correlation between sensor variables and the target variable, and the feature quality assessment table which provides a list of variables with high or moderate predictive value. Note that OD600 is flagged for exclusion due to data leakage.

**FIGURE 10:**
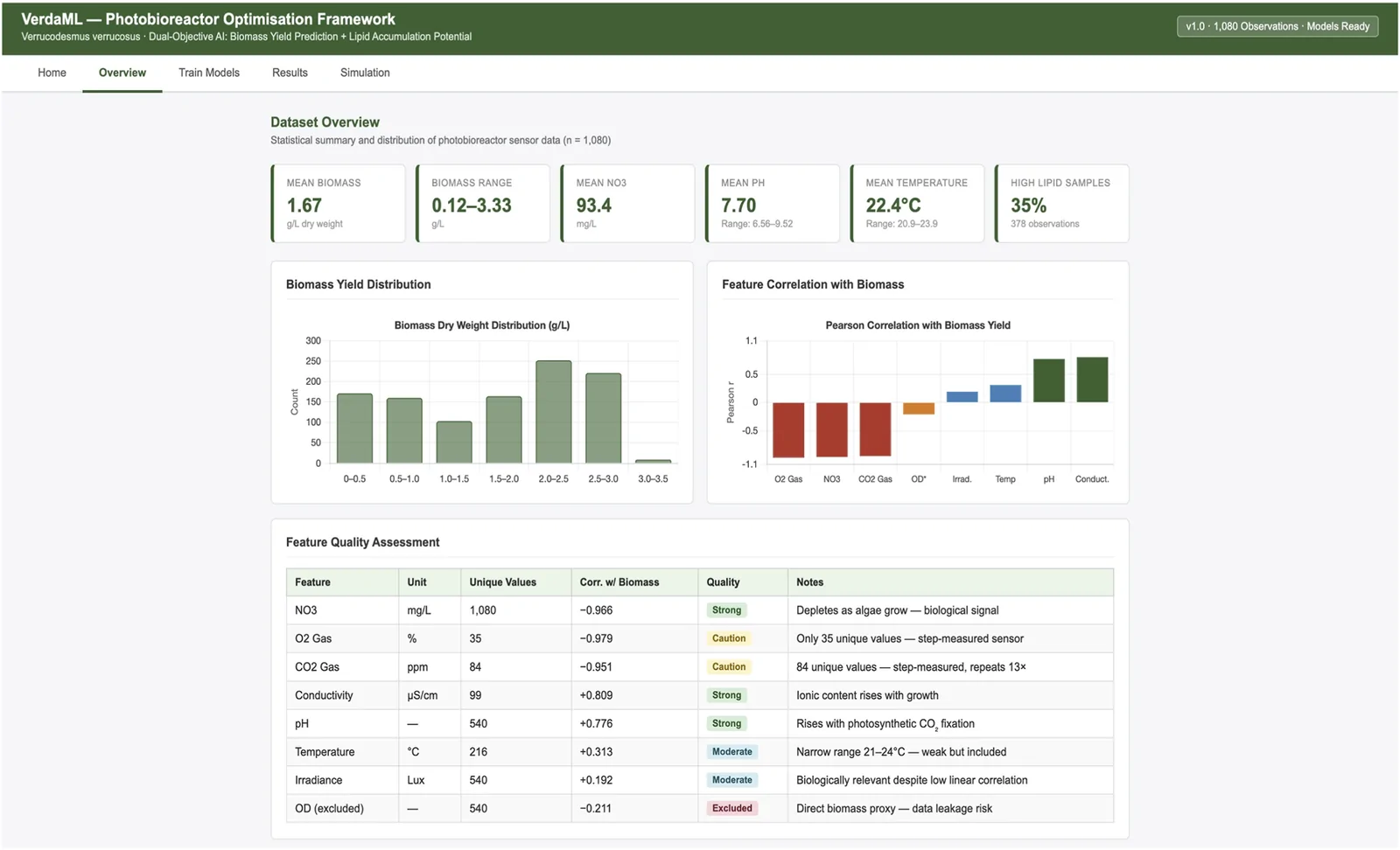
Overview tab showing dataset statistics, biomass yield distribution, sensor variable correlations, and feature quality assessment for the VerdaML photobioreactor framework.

The Results Tab, Figure 11, allows for a comparison of the performance of all regression and classification models. The first is the feature importance bar charts that rank the contribution of all 17 engineered and raw features to predicting the target variable. The second is the *R* ^2^ comparison visualisation, which compares the predicted outcomes from each model.

**FIGURE 11:**
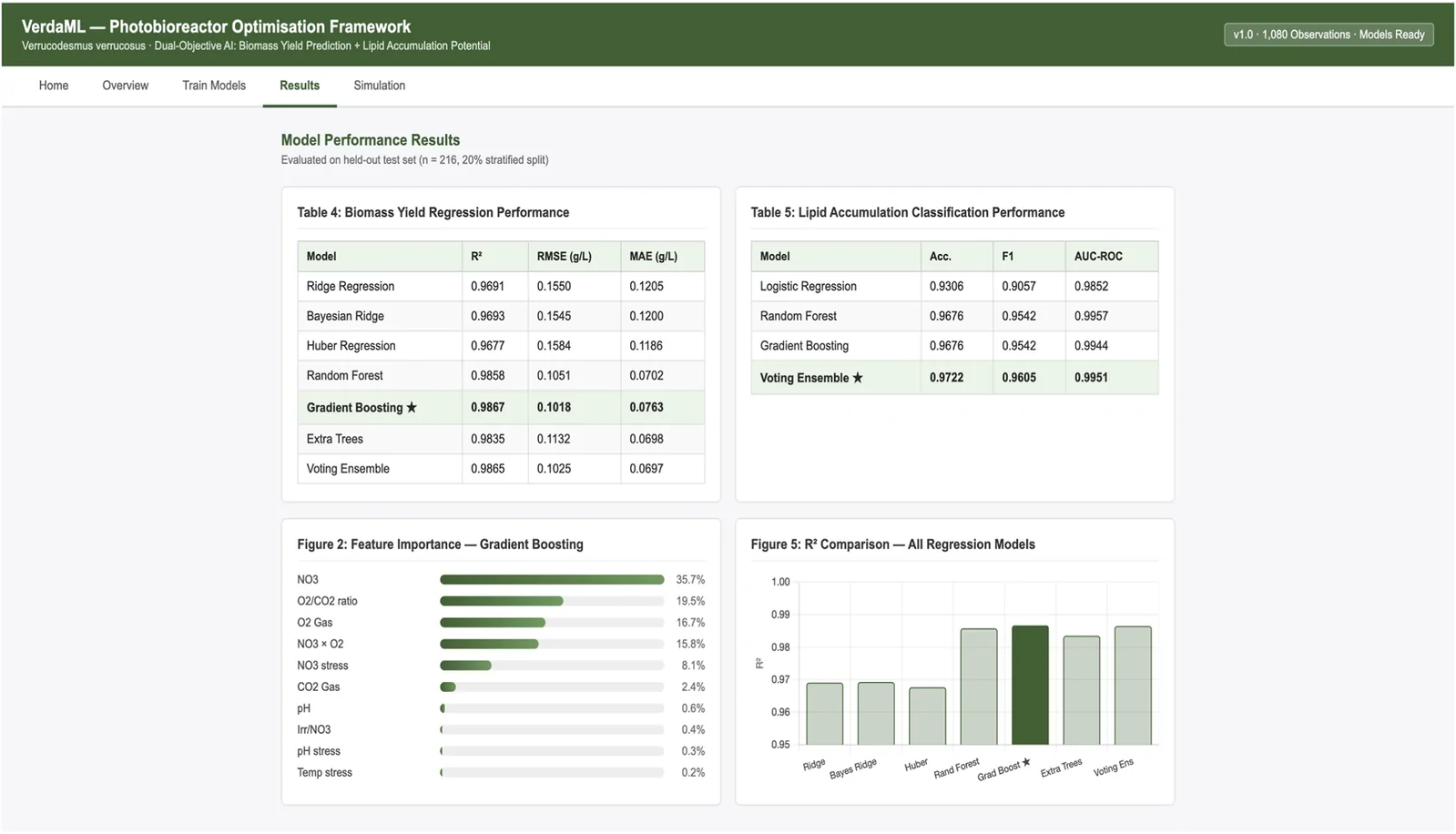
Results tab displaying comparative model performance metrics, feature importance bar chart, and *R* ^2^ comparison visualisation across all regression models.

The Simulation tab, shown in Figures 12, 13 provide an interactive decision-support interface about the operating parameters of the photobioreactor. This interface consists of slider-based controls that allow the user to adjust any of the seven operating parameters of the photobioreactor. The user will receive immediate feedback on both biomass yield and lipid accumulation potential through visually identifying colour-coded indicators. These yield-lipid simulations provide the process engineer with the information necessary to make operating decisions to identify the operating regimes that maximize biomass productivity while also maximizing lipid accumulation. Finding this balance is not possible using single-objective optimization methods.

**FIGURE 12:**
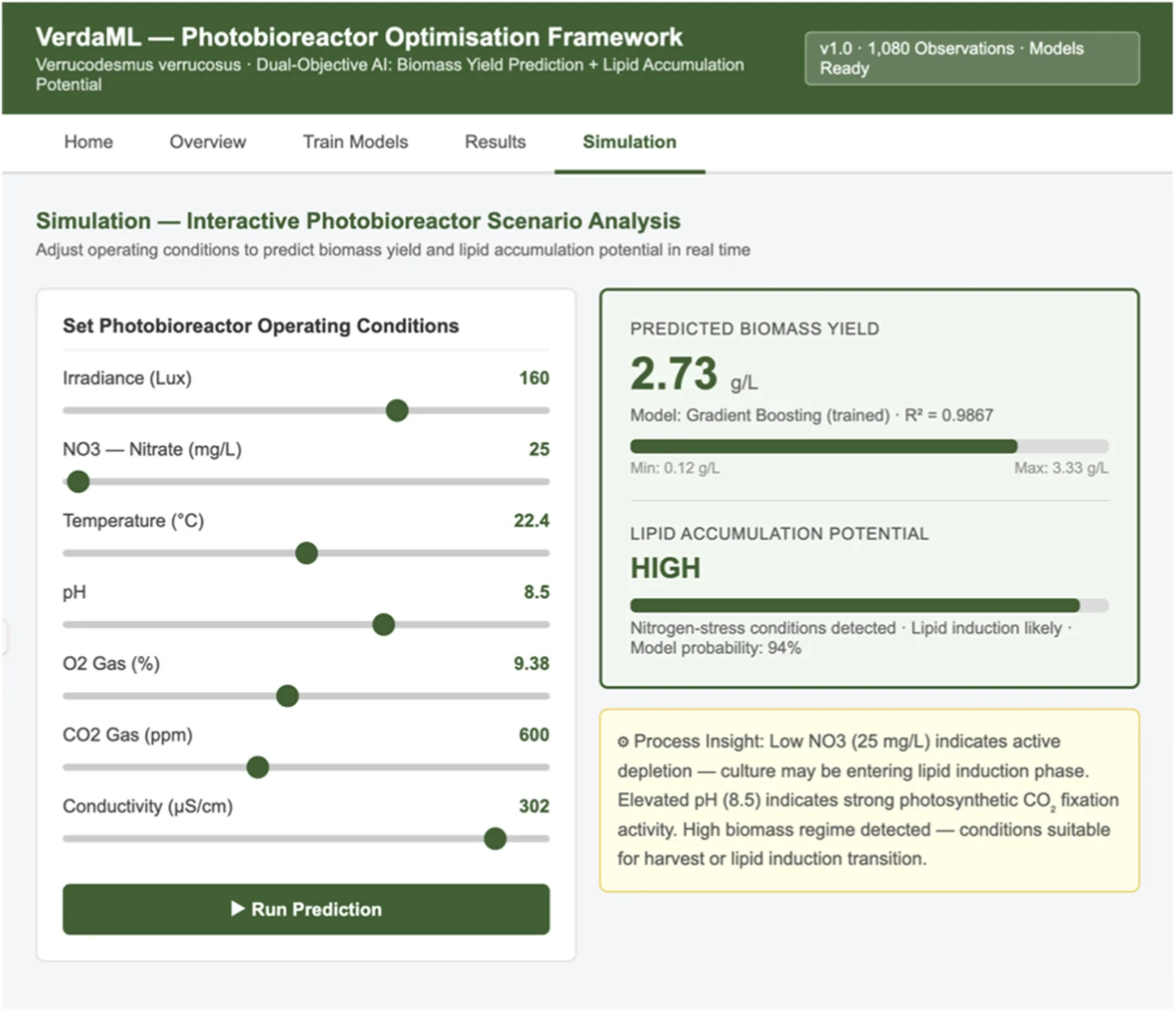
Simulation tab of the VerdaML decision support system showing a high lipid-inductive scenario (NO3 = 25 mg/L, pH = 8.5, Conductivity = 302 μS/cm) with predicted biomass yield of 2.73 g/L and lipid accumulation probability of 94%, consistent with nitrogen-stress conditions expected to favour triacylglyceride biosynthesis.

**FIGURE 13:**
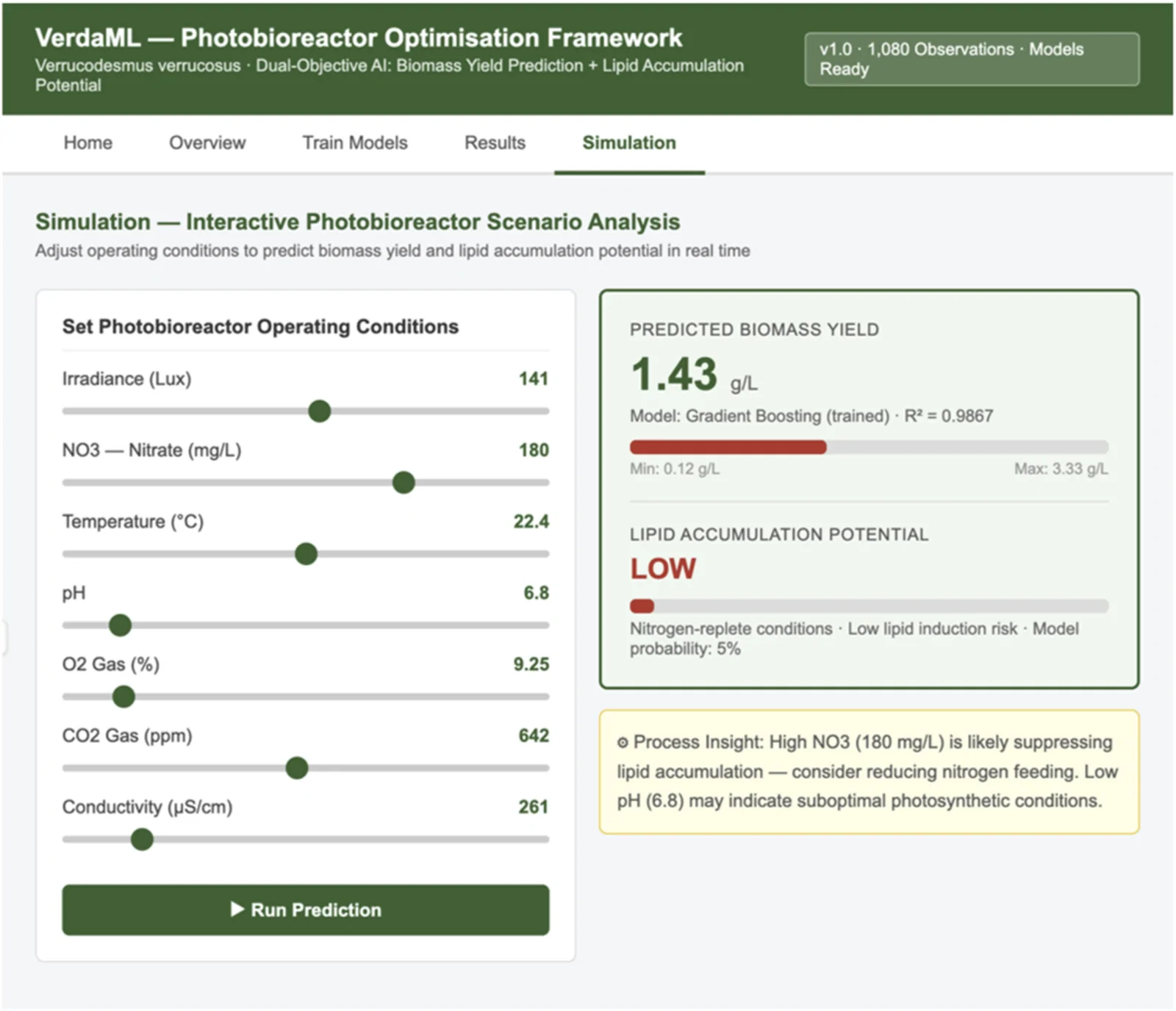
Simulation tab of VerdaML showing a nitrogen-replete scenario (NO3 = 180 mg/L, pH = 6.8, Conductivity = 261 μS/cm) with predicted biomass yield of 1.43 g/L and lipid accumulation probability of 5%, demonstrating the suppression of lipid induction under high nitrogen availability.

The simulations provided results that are highly consistent with the results obtained through the offline evaluation of the model. The predicted outcomes for both yields and lipid potentials were very close to the equivalent test set predictions with the same input conditions.

## 4 Discussion

These results indicate that predictive accuracy in photobioreactor biomass modelling depends more heavily on the quality of feature representation than on model complexity. The Gradient Boosting model (*R* ^2^ = 0.9867) performed very well on the aforementioned data and suggests that the ensemble tree method effectively models the nonlinear interactions between parameters within the biological sensor datasets when used combined with feature engineering based on domain knowledge. Linear models such as Bayesian Ridge and Ridge Regression have *R* ^2^ values greater than 0.969. The engineered feature set significantly linearised the underlying biological growth dynamics of the cultures. Huber Regression produced a viable combination of efficiency for linear models and robustness to outliers for experimental biological data. The ensemble models used in this study demonstrate consistent robustness without requiring additional model complexity that is consistent with the findings from previous studies of bioprocess modelling (Wang et al., 2023; Mukasine et al., 2024).

A central contribution of this study is the integration of nitrogen-stress risk classification with biomass yield prediction within a unified dual-objective framework. Most of the past research in microalgae ML studies attempts to optimise productivity or yield in isolation (Syed et al., 2024; Imamoglu, 2024). The addition of class imbalance correction via SMOTE, combined with ensemble classifiers, produced an AUC-ROC of 0.9903 for lipid potential detection. Ensemble classifiers exhibit significantly higher AUC-ROC than the logistic regression baseline. This supports the notion that there is a nonlinear boundary between the classes in the feature space and therefore tree-based ensemble methodologies are necessary in order to achieve optimal discrimination between the two classes. Together, these results demonstrate the importance of explicitly modelling lipid accumulation potential and also extending beyond yield-only prediction as a key modelling objective (Mondal et al., 2023; Campo-Manzanares et al., 2026).

Clear trade-offs exist between photobioreactor productivity and lipid biosynthesis from the combined risk analyses performed on yield and lipid potential. Photobioreactor operating conditions that favour high biomass and accompany the active growth phase (characterised by an adequate supply of nitrate) are rarely conducive to inducing lipid biosynthesis; instead, lipid accumulation is commonly initiated during nitrogen-limiting conditions in which there is a combination of depleted nitrate and elevated conductivity. High lipid accumulation potential in oleaginous microalgae has been demonstrated to correlate with the above described nitrogen-limiting conditions as well (Hu et al., 2008; Converti et al., 2009). The evidence provided from the trade-off findings indicates the limitations associated with optimising the objectives on a single-objective basis, thus highlighting the value of employing a dual-objective decision making approach for biofuel-oriented microalgal cultivation (Han and Zhang, 2020; Banerjee and Mukhopadhyay, 2023; Brennan and Owende, 2010).

The two most significant predictors of biomass yield were the concentration of nitrates (35.7%) and the engineered O2/CO2 ratio (19.5%). This is biologically consistent—nitrate depletion signals active nitrogen assimilation for biomass synthesis, making it a reliable indirect indicator of culture density and growth phase status (Hu et al., 2008). In contrast, the engineered O2/CO2 gas exchange ratio provides a measure of the net photosynthetic activity, e.g., light availability to the culture, rate of carbon fixation, and metabolic state of the organism, all measured as a single composite variable. These engineered composite features were determined to be statistically more important for prediction than the majority of the individual raw sensor variables, thus validating the approach to domain-driven feature engineering, and underscoring inter-variable encoding within the context of biological ML models (Khaleghi et al., 2021; Mondal et al., 2023).

The advent of VerdaML as an interactive AI-driven, five-tab decision support system significantly increases the ability of the framework to be used in a practical manner. The Simulation tab allows process engineers to model how adjustments to cultivation parameters (e.g., nitrate concentration, irradiance and pH) would affect the operation of their bioreactors in real-time without having to conduct any physical bioreactor experiments, thus reducing the experimental burden of testing multiple variables and accelerating their process development cycles. There is consistently strong agreement between VerdaML predictions and offline evaluations of the test sets, showing that once trained, the predictive fidelity of the data remains intact through interactive deployment. Future closed-loop deployment of VerdaML would require integration of real-time sensor streaming via OPC-UA or MQTT protocols, a Python REST API backend for sub-second inference latency, and a model predictive control layer using VerdaML predictions as the process surrogate. Pilot-scale validation on a 10–50 L photobioreactor with online NO3 and dissolved oxygen sensing would be the next step toward industrial implementation.

## 5 Limitations

Several limitations require consideration. First, both O2 Gas and CO2 Gas sensors only have a limited number of unique values (O2 = 35 and CO2 = 84). This means that these sensors may produce output as stepwise changes rather than continuous output. Additional studies should investigate sensor resolutions and their effect on model performance based on using sensors with greater granularity. Second, only 9 of the observations have biomass concentration levels greater than or equal to 3.0 g/L; therefore, the model accuracy in the high biomass regime is potentially limited because the data set includes a limited number of observations that exceed the average biomass concentration in high-yield regimes. Additional data points that reflect higher productivity ranges would allow for a greater degree of generalization at these higher biomass densities. Third, the target for lipid accumulation potential was determined through operational measurement of biomass concentration due to the lack of available measurement of actual lipid concentration; therefore, it is recommended that additional GC-MS measurement of fatty acid methyl ester profiles be conducted to validate the biological validity of this classification system. Additionally, the VerdaML framework was validated exclusively on Verrucodesmus verrucosus; adaptation to other oleaginous microalgae would require retraining on species-specific datasets and recalibration of the nitrogen-stress threshold to reflect the biomass-lipid relationship of the target organism. Fourth, the Simulation Tab allows for deployment of the fully trained Gradient Boosting Regression Model and Soft Voting Ensemble Classifier directly within VerdaML, with model weights and tree structures exported from the trained sklearn pipeline and set up as native JavaScript inference functions. Therefore, simulation predictions are numerically equal to those obtained in the offline evaluation of the Model, as shown in Tables 4 and 5. Future work will extend the use of VerdaML models to multi-species datasets, in addition to single species datasets, and incorporate timestamped data into an analysis of the cultivation phase to predict outcome. Finally, the feasibility of using these model(s) for closed-loop bioreactor control will be assessed.

## 6 Conclusion

As part of this project, we present the VerdaML framework to sustainably optimise photobioreactor biomass yield and simultaneously determine the nitrogen-stress induction risk through a collection of dual-objective components including predictive modelling for biomass yields, modelling of observed nitrogen-stress induction risk, and an interactive decision-support component. The numerical analyses within VerdaML rely on features created using domain knowledge, an ensemble of machine-learning models, application of SMOTE for correcting imbalanced data, and the use of explanatory methods to inform the user about why the selected solutions were chosen for an optimal cultivation scenario. By combining predictors of biomass yield to allow for maximum productivity, and modelling of observed nitrogen-stress induction risk with the intent of using these data to maximise lipid induction, VerdaML directly addresses two key challenges associated with optimising productivity and effectively managing the induction of lipid synthesis for the production of microalgal biofuels. The results demonstrate that Gradient Boosting Regression models have produced nearly ideal biomass predictions (*R* ^2^ = 0.9867) and Soft Voting Ensemble classifiers achieved an AUC-ROC of 0.9903 with respect to nitrogen-stress induction risk classification, thus providing the first published benchmarks associated with machine learning for this species of microalgae. Five-fold cross-validation confirmed model generalisability, with Gradient Boosting achieving a mean cross-validated *R* ^2^ of 0.9842 (SD = 0.0024), consistent with the held-out test performance. Additionally, the feature importance analyses indicated that the principal drivers of performance are the depletion of nitrate and the ratio of O2 and CO2 exchanged throughout the photobioreactor. The analyses further identified a clear trade-off between biomass productivity and lipid biosynthesis, thus indicating that to optimise cultivation for biofuels, single-objective optimisation of algal cultivation is not an appropriate approach (Han and Zhang, 2020; Banerjee and Mukhopadhyay, 2023; Brennan and Owende, 2010). The VerdaML decision support system provides an interactive AI-driven tool with the capability of performing real-time simulations of various photobioreactor processes.

Future work will include integration of live sensor streaming for closed-loop photobioreactor control, expanding the use of the framework to more than one species as well as examining data collected from pilot studies, and using reinforcement learning techniques to manage the dynamic phases of cultivation. VerdaML provides a basis for the development of an AI-driven framework of optimising microalgal bioprocesses that are scalable, explainable, and able to be applied in practice to aid in sustainable biofuel production; this is in alignment with UN Sustainable Development Goals 7 and 9.

## Data Availability

The original contributions presented in the study are included in the article/supplementary material, further inquiries can be directed to the corresponding authors.

## References

[B1] Abiusi F. Wijffels R. H. Janssen M. (2014). Light-limited growth of Chlorella sorokiniana UTEX 1230. Biotechnol. Bioeng. 111 (5), 927–936. 10.3390/biology7020025 24895246

[B2] Aziz M. M. A. Elgammal E. W. Ghitas R. G. (2020). Comparative study on modelling by neural networks and response surface methodology for better prediction and optimisation of fermentation parameters. Biocatal. Agric. Biotechnol. 25, 101619. 10.1016/j.bcab.2020.101619

[B3] Banerjee D. Mukhopadhyay A. (2023). Perspectives in growth production trade-off in microbial bioproduction. RSC Sustain. 1 (2), 224–233. 10.1039/D2SU00066K

[B4] Brennan L. Owende P. (2010). Biofuels from microalgae — a review of technologies for production, processing, and extractions of biofuels and co-products. Renew. Sustain. Energy Rev. 14 (2), 557–577. 10.1016/j.rser.2009.10.009

[B5] Breuer G. Lamers P. P. Martens D. E. Draaisma R. B. Wijffels R. H. (2012). The impact of nitrogen starvation on the dynamics of triacylglycerol accumulation in nine microalgae strains. Bioresour. Technol. 124, 217–226. 10.1016/j.biortech.2012.08.003 22995162

[B6] Campo-Manzanares N. Moimenta A. R. Balsa-Canto E. (2026). Critical assessment of machine learning approaches for classification, dynamic prediction and surrogate modelling in food fermentation. Food Res. Int. 229, 118403. 10.1016/j.foodres.2026.118403 41763759

[B7] Chawla N. V. Bowyer K. W. Hall L. O. Kegelmeyer W. P. (2002). SMOTE: synthetic minority over-sampling technique. J. Artif. Intell. Res. 16, 321–357. 10.1613/jair.953

[B8] Chisti Y. (2007). Biodiesel from microalgae. Biotechnol. Adv. 25 (3), 294–306. 10.1016/j.biotechadv.2007.02.001 17350212

[B9] Converti A. Casazza A. A. Ortiz E. Y. Perego P. Del Borghi M. (2009). Effect of temperature and nitrogen concentration on the growth and lipid content of *Nannochloropsis oculata* and *Chlorella vulgaris* for biodiesel production. Chem. Eng. Process. 48 (6), 1146–1151. 10.1016/j.cep.2009.03.006

[B10] De Farias Silva C. E. Costa G. Y. S. C. M. Ferro J. V. de Oliveira Carvalho F. da Gama B. M. V. Meili L. (2022). Application of machine learning to predict the yield of alginate lyase solid-state fermentation by *cunninghamella echinulata*: artificial neural networks and support vector machine. Reac Kinet. Mech. Cat. 135, 3155–3171. 10.1007/s11144-022-02293-9

[B11] del Rio-Chanona E. A. Wagner J. Ali H. Fiorelli F. Zhang D. Hellgardt K. (2019). Deep learning-based surrogate modeling and optimization for microalgal biofuel production and photobioreactor design. AIChE J. 65 (3), 915–923. 10.1002/aic.16473

[B12] Han Y. Zhang F. (2020). Control strategies to manage trade-offs during microbial production. Curr. Opin. Biotechnol. 66, 158–164. 10.1016/j.copbio.2020.07.004 32810759 PMC8021483

[B13] Hernandez-de Leon H. R. Casanova-Pelaez P. J. Cruz-Fuentes A. A. Trujillo-de Santiago G. Morales-Oyervides L. Fajardo-Diaz J. L. (2025). A comprehensive dataset of biomass and critical variables for *Verrucodesmus verrucosus* culture in bubble column photobioreactors. Data Brief 61, 112003. 10.1016/j.dib.2025.112003 PMC12452730 40989232

[B14] Hu Q. Sommerfeld M. Jarvis E. Ghirardi M. Posewitz M. Seibert M. (2008). Microalgal triacylglycerols as feedstocks for biofuel production: perspectives and advances. Plant J. 54 (4), 621–639. 10.1111/j.1365-313X.2008.03492.x 18476868

[B15] Igou T. Zhong S. Reid E. Chen Y. (2023). Real-time sensor data profile-based deep learning method applied to open raceway pond microalgal productivity prediction. Environ. Sci. and Technol. 57 (22), 8246–8256. 10.1021/acs.est.2c07578 37234045 PMC10666538

[B16] Imamoglu E. (2024). Artificial intelligence and/or machine learning algorithms in microalgae bioprocesses. Bioengineering 11 (11), 1143. 10.3390/bioengineering11111143 39593803 PMC11592280

[B17] Juneja A. Ceballos R. M. Murthy G. S. (2013). Effects of environmental factors and nutrient availability on the biochemical composition of algae for biofuels production. Energies 6 (9), 4607–4638. 10.3390/en6094607

[B18] Khaleghi M. K. Savizi I. S. P. Lewis N. E. Shojaosadati S. A. (2021). Synergisms of machine learning and constraint-based modeling of metabolism for analysis and optimization of fermentation parameters. Biotechnol. J. 16 (11), 2100212. 10.1002/biot.202100212 34390201

[B19] Klok A. J. Martens D. E. Wijffels R. H. Lamers P. P. (2013). Simultaneous growth and neutral lipid accumulation in microalgae. Bioresour. Technol. 134, 233–243. 10.1016/j.biortech.2013.02.006 23500580

[B20] Mata T. M. Martins A. A. Caetano N. S. (2010). Microalgae for biodiesel production and other applications: a review. Renew. Sustain. Energy Rev. 14 (1), 217–232. 10.1016/j.rser.2009.07.020

[B21] Mondal P. P. Galodha A. Verma V. K. Singh V. Show P. L. Awasthi M. K. (2023). Review on machine learning-based bioprocess optimization, monitoring, and control systems. Bioresour. Technol. 370, 128523. 10.1016/j.biortech.2022.128523 36565820

[B22] Mukasine A. Sibomana L. Jayavel K. Nkurikiyeyezu K. Hitimana E. (2024). Maximizing biogas yield using an optimized stacking ensemble machine learning approach. Energies 17 (2), 364. 10.3390/en17020364

[B23] Park S. Y. Park C. H. Choi D. H. Hong J. K. Lee D. Y. (2021). Bioprocess digital twins of mammalian cell culture for advanced biomanufacturing. Curr. Opin. Chem. Eng. 33, 100702. 10.1016/j.coche.2021.100702

[B24] Posten C. (2009). Design principles of photo-bioreactors for cultivation of microalgae. Eng. Life Sci. 9 (3), 165–177. 10.1002/elsc.200900003

[B25] Quinn J. C. Davis R. (2015). The potentials and challenges of algae based biofuels: a review of the techno-economic, life cycle, and resource assessment modeling. Bioresour. Technol. 184, 444–452. 10.1016/j.biortech.2014.10.075 25453439

[B26] Ramchuran S. O. O'Brien F. Dube N. Ramdas V. (2023). An overview of green processes and technologies, biobased chemicals and products for industrial applications. Curr. Opin. Green Sustain. Chem. 41, 100832. 10.1016/j.cogsc.2023.100832

[B27] Rashedi M. Khodabandehlou H. Demers M. Wang T. Garvin C. (2022). Model predictive controller design for bioprocesses based on machine learning algorithms. IFAC-PapersOnLine 55 (7), 45–50. 10.1016/j.ifacol.2022.07.420

[B28] Syed T. Krujatz F. Ihadjadene Y. Mühlstädt G. Hamedi H. Mädler J. (2024). A review on machine learning approaches for microalgae cultivation systems. Comput. Biol. Med. 172, 108248. 10.1016/j.compbiomed.2024.108248 38493599

[B29] Wang Z. Peng X. Xia A. Shah A. A. Huang Y. Zhu X. (2022). The role of machine learning to boost the bioenergy and biofuels conversion. Bioresour. Technol. 343, 126099. 10.1016/j.biortech.2021.126099 34626766

[B30] Wang Y. Zhou J. Wang X. Yu Q. Sun Y. Li Y. (2023). Rumen fermentation parameters prediction model for dairy cows using a stacking ensemble learning method. Animals 13 (4), 678. 10.3390/ani13040678 36830465 PMC9951746

[B31] Wu Y. Li Z. Liu Z. Huang J. (2025). Harnessing artificial intelligence to revolutionize microalgae biotechnology. Mar. Drugs 23 (5), 184. 10.3390/md23050184 40422774 PMC12113173

[B32] Yee C. S. Zahia-Azizan N. A. Abd Rahim M. H. Mohd Zaini N. A. Raja-Razali R. B. Ushidee-Radzi M. A. (2025). Smart fermentation technologies: microbial process control in traditional fermented foods. Fermentation 11 (6), 323. 10.3390/fermentation11060323

